# Municipal Milk Supply

**Published:** 1904-02-20

**Authors:** 


### Editor's Letter-Box.

[Our Correspondents are reminded that prolixity is a great bar to publication, and that brevity of style and conciseness of statement greatly facilitate early insertion.]

MUNICIPAL MILK SUPPLY.

The Secretary of the East London Hospital for Children writes:—As public attention has lately been drawn by you to the subject of municipal milk supply it may perhaps interest you and your readers to learn that the board of this hospital has recently appointed a committee to consider the question of the establishment of a depot, under the control of the hospital, for the distribution of milk, either humanised, sterilised, or pasteurised as may be decided, for the use of infants attending the out-patient department, or under the care of local medical practitioners.

The improper feeding of infants is a difficulty that the medical staff of the hospital have always had to contend with, and it is felt that the time has arrived when steps should be taken to remedy the evil. Of course none of the funds of the hospital can be diverted to the founding of this depot, although once it is working the custom of the hospital will be, in a measure, a guarantee of its continued success. Consequently a subscription list has been opened to cover the initial expenses of adapting premises and installing appropriate equipment, estimated at £450 to £500.

A letter from Colonel Needham, the chairman of the board of management of the hospital, published in the *Morning Post*, February 5th last, more fully sets out the aims and needs of the proposed depot.

